# Research on the frailty status and adverse outcomes of elderly patients with multimorbidity

**DOI:** 10.1186/s12877-022-03194-1

**Published:** 2022-07-06

**Authors:** Jing Lv, Rao Li, Li Yuan, Xiao-ling Yang, Yi Wang, Zi-Wei Ye, Feng-Mei Huang

**Affiliations:** 1 West China School of Nursing/ West China Hospital Endocrinology and Metabolism Department, Sichuan University, Chengdu, 610041 People’s Republic of China; 2 West China Hospital Endocrinology and Metabolism Department/ West China School of Nursing, Sichuan University, Chengdu, 610041 People’s Republic of China

**Keywords:** Elderly, Chronic diseases, Multimorbidity, Frailty, Adverse outcomes, Risk factors

## Abstract

**Background:**

As patients age, the frailty of those with multimorbidity increases, often resulting in adverse health outcomes. The current study investigated the frailty status and the factors which influence it in elderly patients with multimorbidity in Chinese hospitals. The relationship between the frailty of patients with multimorbidity and adverse outcomes was explored.

**Methods:**

The current prospective cohort study investigated inpatients in the internal medicine department of 5 tertiary hospitals in Sichuan Province, China. A total of 3836 elderly patients with multimorbidity were enrolled. Frailty was assessed using the FRAIL scale and adverse outcome events occurring during hospitalization were tracked. Descriptive statistics and logistic regressions were used for data analysis.

**Results:**

The prevalence of frailty was 27.2% and of pre-frailty, 58.9%. Logistic regression analysis showed that increasing age, low BMI, low education level, lack of exercise, multiple types of medications and multiple numbers of chronic diseases were the main risk factors for frailty in elderly patients with multimorbidity (OR values: 1.020, 1.469, 2.350, 2.836, 1.156 and 1.308, respectively). The incidence of adverse outcomes was 13.9% among the cohort with the most common being deep vein thrombosis (42.4%), followed by pressure injury (38.8%). Regression analysis showed a significant correlation of frailty with adverse outcome (OR: 1.496; *p* < 0.01).

**Conclusions:**

The prevalence of frailty and pre-frailty in hospitalized elderly patients with multimorbidity was high. Increasing age, low BMI, low education level, lack of exercise, multiple types of medications and multiple numbers of chronic diseases were factors which influenced frailty and frailty was an important factor in the occurrence of adverse outcomes. The most common adverse outcome of elderly multimorbidity patients during hospitalization was deep vein thrombosis.

## Introduction

With age, older people tend to accumulate multiple chronic diseases; this condition is referred to as “multimorbidity” (having two or more chronic diseases) [1]. It has been suggested that the prevalence of multimorbidity among people aged 65–84 years is 64.9% globally [2] and the proportion of the Chinese population over 60 years with two or more chronic diseases may reach 76.5%. Among those over 80 years, a higher proportion (80%) may be affected [3]. Chronic multimorbidity in the elderly are a common phenomenon, presenting a serious challenge for medical and health workers. Meanwhile, multimorbidity have become a significant indicator for monitoring the health of elderly individuals [4].

Frailty is often defined as a clinical syndrome driven by age-related biologic changes that drive physical characteristics of frailty and eventually, adverse outcomes [5]. There are currently many definitions and assessment tools for frailty, such as the frailty phenotype proposed by Fried and Walston, which defined frailty as "a biologic syndrome of decreased reserves and resistance to stressors, resulting from cumulative declines across multiple physiological systems and causing vulnerability to adverse outcomes" [6]. Its main pathophysiological characteristics are thought to be oxidative stress and chronic inflammation [7, 8]. The accumulation of deficits definition of frailty, commonly referred to as the frailty index (FI) is based on the cumulative effect of medical, functional and psychosocial age-related deficits [9]. In addition, the International Association for Nutrition and Aging proposed a frailty scale (FRAIL) that requires only 5 simple questions to be answered, include self-reported fatigue, resistance, ambulation, illness and Loss of weight. Increased recognition of frailty without face-to-face examination [10]. Multinational epidemiological surveys have shown the global nature of frailty which has a prevalence of between 12 and 24% among community dwelling older adults [11]. Multimorbidity interact with frailty, and patients with chronic multimorbidity are more likely to develop frailty than the general elderly [12]. The occurrence and development of multimorbidity and frailty are closely related in geriatric medicine and are risk factors for adverse outcomes in elderly hospitalized patients [13]. Worsening of the multimorbidity state contributes to frailty effects and individuals are more likely to experience adverse outcomes. There is a two-fold increase in risk of falls and fractures, physical impairment, loss of independence in daily activities, hospitalization and death [14, 15].

Few comprehensive assessments and analyses have been conducted regarding the frailty of elderly Chinese patients with multimorbidity with most previous studies having focused on a single component of frailty. However, there remains a pressing need to evaluate the impact of frailty and multimorbidity in elderly patients and to assess possibilities for intervention. The current study used a multicenter large sample survey to assess frailty status in hospitalized elderly patients with multimorbidity. The impact of frailty status on clinical adverse outcomes was evaluated. The aim was to make medical workers more aware of the need to assess the frailty of elderly patients with multimorbidity and provide theoretical support for timely screening and formulation of intervention measures. Moreover, we aimed to link multimorbidity-frailty-adverse outcome events in hospitalized patients to provide a reference for future exploration of these relationships and a basis for preventive interventions for clinical adverse events.

## Methods

### Study design and patient recruitment

**Fig. 1:**
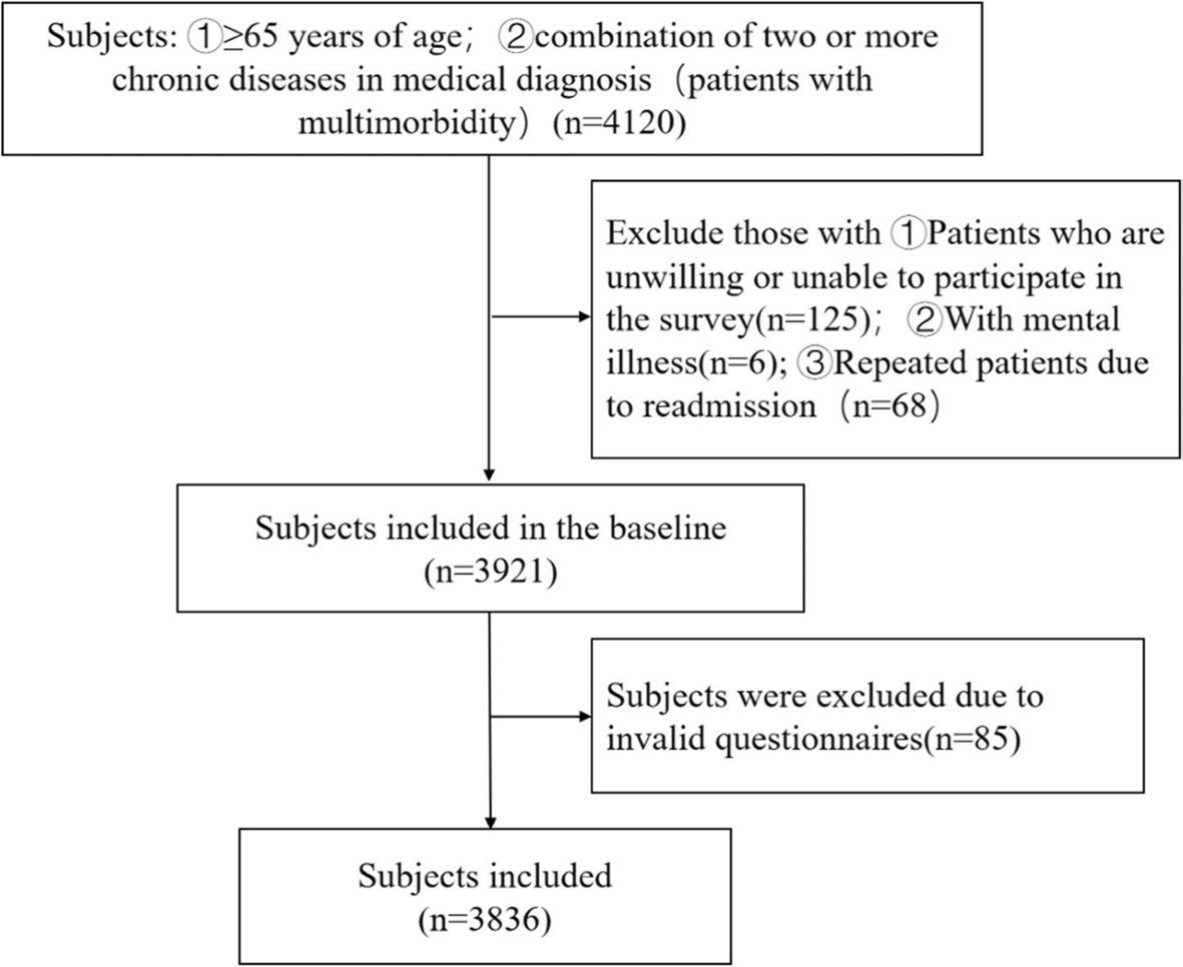
Flow chart of study participants

The current prospective cohort study used convenience sampling to enroll elderly patients with chronic multimorbidity who were hospitalized in the internal medicine department of five tertiary general hospitals in Sichuan Province between March 2020 and July 2021. The inclusion criteria were as follows: 1) ≥ 65 years of age; 2) diagnosis of 2 or more chronic diseases according to the 12 chronic diseases specified in the Tenth Revision of the International Statistical Classification of Diseases and Related Health Problems (ICD-10) [16]. Patients with severe mental illness, who were unwilling or unable to participate in the survey, or who had repeated admissions were excluded. A total of 3836 patients were enrolled (Fig. 1). All interviews were conducted by trained examiners. Data collection and frailty assessment were performed within 3 days of admission and patients were followed up for adverse outcome events during the hospital stay. The current study was approved by the regional ethics committee.

### General information questionnaire

A general information questionnaire was designed by researchers after consultation with literature experts. Information sought included: gender (female: 0 and male: 1), age (years), body mass index (BMI, calculated from weight and height: < 24: 0, ≥ 24: 1) and educational status (primary and below: 0, secondary: 1, college and above: 2), marital status (unmarried, divorced or widowed: 0; married: 1), living style (living alone: 0, living with family members: 1), monthly income (≤ 2000 yuan: 0, 2000–5000 yuan: 1, ≥ 5000 yuan: 2), medical insurance status (no medical insurance: 0, medical insurance: 1), smoking (never smoked: 0, current/former smokers: 1), drinking (teetotal: 0, drinker: 1), exercise (no: 0, yes: 1). Age was regarded as continuous variable and other data were processed according to classification and coding. In addition, intubation status, such as PICC, CVC, urinary catheter, gastric tube, tracheal intubation and various wound drainage tubes, was included (no tube: 0, tube: 1).

### Medication status

Prescribed drugs were divided into categories as follows: respiratory system, digestive system, urinary system, blood system, antihypertensive, lipid-lowering, antiarrhythmic, hypoglycemic, anticoagulants, sedatives and sleeping pills. All numbers of drug types were included in the analysis as a continuous variable.

### Definition of multimorbidity

The multimorbidity is defined as "the simultaneous presence of two or more chronic diseases" [1]. The 12 diseases defined by the tenth revision of the International Statistical Classification of Diseases and Related Health Problems (ICD-10) were assessed by the current study and included as a continuous variable. These are hypertension, diabetes, coronary heart disease, chronic heart failure, chronic obstructive pulmonary disease, cerebrovascular disease, bone and joint disease, chronic kidney disease, chronic gastrointestinal disease, chronic liver disease, cancer and depression [16].

### Frailty assessment

Frailty status was assessed using the self-assessment FRAIL scale, proposed by the International Association of Nutrition and Aging (IANA) in 2008 [10, 17]. The FRAIL scale includes 5 components: self-reported fatigue (feeling tired most or all of the time in the past 4 weeks); resistance (difficulty walking up 10 steps alone without resting and without aids); ambulation (difficulty walking several hundred yards alone and without aids); illnesses (suffering from more than 5 diseases), weight loss (weight decline of 5% or greater within the past 12 months) [10]. Each criterion is awarded one point and total sores are assessed as follows: 0: robust; 1–2: pre-frailty; 3–5: frailty. The current study regarded the occurrence of frailty as a binary variable: 0: no frailty (robust and pre-frailty); 1: frailty.

### Assessment of adverse outcomes

Adverse outcome events were based on China's "Medical Quality Control Indicators for Nursing Professionals (2020 Edition)" [18] and risk events with high incidence and serious impact were selected as observation indicators. Events include: (1) Falls: a sudden and involuntary position change of participants during hospitalization, falling to the ground or a lower plane, including falling from bed; (2) Pressure injury: According to the "Prevention and Treatment of Pressure Ulcers/Injuries: Clinical practice guideline" published in 2019 [19], pressure injury in this study was defined as: new onset pressure injury of grade I or above after admission, including grade I to IV, unstageable pressure injury and suspected deep tissue pressure injury; (3) Deep vein thrombosis (DVT): DVT was diagnosed by Doppler ultrasonography during hospitalization without a diagnosis of DVT at admission. (4) Cardiac arrest: refers to the cardiac arrest events that occurred before participants were discharged from the hospital. (5) Aspiration: sluggish swallowing reflex due to food stuck in the throat or entering the trachea or esophagus, ventilation disorder and asphyxia during hospitalization; (6) Unplanned extubation: for extubation that occurred during the hospitalization of the research subjects outside the scope of the diagnosis and treatment plan, including accidental catheter detachment or self-extubation, premature extubation due to catheter quality problems, catheter blockage and catheter-related infection; (7) Lost: research subjects who ran away or disappeared for various reasons without the consent of medical staff during hospitalization. The occurrence of adverse outcomes was a dichotomous variable: 0: no adverse outcome: 1: adverse outcome.

### Statistical analysis

SPSS software for Windows Version 25 (SPSS Inc., Chicago, IL) was used for statistical analyses. Statistical tests and confidence intervals were two-sided with a significance level of *p* < 0.05. The chi-square test was used for comparison of categorical variables. Independent sample t-tests were used for normally distributed continuous variables and two-independent sample Mann–Whitney U test for non-normally distributed variables. Relationships between comorbidities and frailty and between frailty and adverse outcomes were analyzed by logistical regression.

## Results

### Sociodemographic characteristics of the participants

A total of 3836 older adults with multimorbidity were recruited. The age range was 65–96 years old (mean = 73.78 ± 6.451), 2168 were male (56.5%), 2504 had BMI < 24 (65.3%). Educational status was as follows: illiterate or primary school only: 2062 (53.7%); secondary school: 1387 (36.1%) and high school or above: 383 (10%). Married participants numbered 3,231 (84.2%) and 3,593 (93.6%) were living with their families. There were 507 patients who smoked (13.2%); 408 who consumed alcohol (10.6%) and 2171 (56.6%) who exercised. Income was stratified as 1975 (51.5%) who earned 2000 ~ 5000 yuan/month and 3435 had medical insurance (89.5%). A total of 824 (21.5%) were intubated.

The number of diseases per patient ranged from 2–8 with the most common being hypertension and diabetes, and 29.5% had both these diseases (Table 1). A total of 1794 patients (46.8%) had 2 types of chronic disease; 1276 (33.3%) had 3 types, 525 (13.7%) had 4 types and 241 (6.28%) had 5 types or more.

**Table 1:** Prevalence of elderly patients with multimorbidity

Variables	Frequency	Percentage (%)	Variables	Frequency	Percentage (%)
Hypertension	2530	66.0%	CKD	692	18.0%
Diabetes	1652	43.1%	CGD	685	22.4%
CHD	1160	30.2%	CLD	323	8.45%
CHF	417	10.9%	Cancer	605	15.8%
COPD	985	25.7%	Depression	47	1.22%
CVD	859	22.4%	KOA	727	19.0%

*Abbreviations*: *CHD* Coronary Heart Disease, *CHF* Chronic heart failure, *COPD* Chronic obstructive pulmonary disease, *CVD* Cerebral vascular diseases, *CKD* Chronic kidney disease, *CGD* Chronic gastrointestinal disease, *CLD* Chronic liver disease, *KOA* Knee osteoarthritis

### Frailty status and contributory factors

Of the cohort, 1043 patients were diagnosed with frailty, according to the FRAIL scale, giving a prevalence of 27.2% and 2258 patients were diagnosed as pre-frail (58.9%).

Univariate analysis showed that age, BMI, marital status, educational level, life status, exercise, income, number of chronic diseases and number of drug types were significantly different among the frail and non-frail patients (*p* < 0.05).

Taking the frail group as the dependent variable, the statistically significant age, BMI, marital, life, educational status, exercise, income, number of chronic diseases and number of drug types were the independent variables for multivariate logistical regression analysis. Increased age, low BMI, low educational status, lack of exercise, increased numbers of drug types and increased numbers of chronic diseases were the influencing factors of frailty in elderly patients with multimorbidity (OR: 1.020, 1.469, 2.350, 2.836, 1.156 and 1.308, respectively) (Table 2).

**Table 2:** Logistic regression analysis of predictors of frailty

Variables	β	SE	Wald2	P	OR	95% CI
Age	0.019	0.006	9.651	0.002	1.020	1.019–1.007
BMI	0.385	0.085	20.534	< 0.001	1.469	1.243–1.737
Education	0.854	0.149	32.734	< 0.001	2.350	1.754–3.149
Exercise	1.042	0.079	172.333	< 0.001	2.836	2.427–3.314
Number of drug types	0.145	0.023	39.650	< 0.001	1.156	1.105–1.209
Number of chronic diseases	0.268	0.040	44.343	< 0.001	1.308	1.209–1.415
Constant	-5.354	0.540	98.220	< 0.001	0.005	

### The relationship between frailty and adverse outcomes

A total of 533 adverse outcomes occurred, affecting 13.9% (533/3836) of participants, of which 41 were cases of falls, 202 were cases of pressure injury, 221 were cases of deep vein thrombosis, 54 were cases of cardiac arrest, 13 were cases of lost, 2 were cases of aspiration and 4 were cases of unplanned extubation.

Binary logistical regression analysis showed that, after adjusting for sociodemographic factors, medication status and disease status as covariates, there was a significant positive relationship between frailty and adverse outcomes (OR:1.496; *p* < 0.01). Additionally, lack of exercise, no medical insurance, increased numbers of drug types and the presence of intubation were risk factors against the adverse outcomes (OR: 1.986, 2.528, 1.195 and 3.374, respectively) (Table 3).

**Table 3:** Logistic regression analysis of factors affecting adverse outcomes

Variables	β	SE	Wald2	P	OR (95%CI)
Numbers of drug types	0.178	0.028	41.447	< 0.001	1.195 (1.132–1.261)
Intubation status	1.216	0.103	139.617	< 0.001	3.374 (2.758–4.128)
Exercise	0.686	0.104	43.127	< 0.001	1.986 (1.618–2.437)
Medical insurance	0.928	0.133	48.356	< 0.001	2.528 (1.947–3.284)
Frailty	0.403	0.108	13.908	< 0.001	1.496 (1.211–1.849)
Constant	-4.526	0.563	64.557	< 0.001	0.011

## Discussion

The current study found that common diseases of hospitalized elderly patients with multimorbidity are hypertension, diabetes and coronary heart disease. The most common chronic disease was hypertension and diabetes and 29.5% of participants had both these diseases. These findings are consistent with those of Skinner and Woods et al.who found that 2–3 chronic diseases are most common in the elderly and that these include cardiovascular disease and diabetes [20, 21]. Multimorbidity have become the main challenges facing health systems worldwide. It has an adverse multiplicative effect on all health outcomes [22]. Frailty is also a common clinical indicator of poor prognosis in the elderly and a bidirectional causal relationship between them is probable. The incidence of frailty in elderly Chinese individuals is thought to range from 9.9% to 31.92% and pre-frailty from 33.9–39.62% [23–25]. Elsewhere, studies have reported frailty and pre-frailty rates to be 16%-94% and 38%-87.8%, respectively, among geriatric patients [4, 13, 26]. The incidence of frailty in hospitalized elderly patients with multimorbidity was 27.2% and of pre-frailty, 58.9%, among the patients of the current study. There is a current lack of uniformity in the definition and quantification of frailty and different scales and basic concepts are used, a situation which has resulted in a wide range of prevalence rates for frailty and pre-frailty being reported. In addition, differences in study populations also affect reported results. Although there are differences in the prevalence of frailty reported by various studies, the harmful effects to the elderly population are generally acknowledged. Early recognition of frailty and appropriate intervention can help to reduce the occurrence of adverse events.

Demographic characteristics have a substantial impact on the occurrence of frailty in elderly hospitalized patients. Increased age and low BMI are particularly potent factors and, in this respect, the findings of the current study were in agreement with those of Kshatri [27]. Low educational status was also a risk factor, perhaps because people with a higher educational level usually have better living conditions, are more aware of their own health care and pay more attention to disease prevention. Previous studies indicate that regular exercise improves muscle strength, physical fitness and metabolism in prefrail elderly individuals. Indeed, 12 weeks of resistance training for the lower limbs of elderly patients was found to delay aging and enhance physical condition in pre-frail elderly individuals [28]. Clinical staff should formulate individualized frailty risk assessments and arrange aerobic and resistance training for pre-frail and frail patients to strengthen muscles [29].

The current study has also established a link between the number of chronic diseases, number of drug types and the risk of frailty (OR: 1.308; 95% CI: 1.209–1.415; OR: 1.156; 95% CI 1.105–1.209, respectively). Previous studies have established links between a single disease and frailty. For example, frailty prevalence in patients with acute decompensated heart failure was as high as 98% [30, 31] and that for patients with COPD was twice that of those without [32, 33]. Interactions between diseases in multimorbidity patients and interactions with therapeutic drug combinations may increase the risk of frailty. The British Biological Database [21] showed that patients with 4 or more chronic diseases had a significantly increased risk of frailty (OR: 27.1; 95% CI: 25.3–29.1). Factors such as drug-drug interactions, drug-disease interactions, potential adverse effects, drug pharmacokinetics and pharmacodynamics, toxicity and therapeutic effects may all contribute to the development of frailty [34]. Gnjidic and Hilmer et al*.* indicated that some frailty clinical effects are directly related to the number of drugs taken and these include weight loss, balance disorders, poor nutritional status and deterioration of function [35–37]. Another study found an association between anticholinergic drugs and the occurrence of frailty [38]. The relationship between drugs and frailty is complicated by the fact that elderly patients seem to be more sensitive to certain drugs and multiple drug-drug interactions may influence the frailty rate [39]. It can be inferred that clinical staff should carefully scrutinize the medication of elderly patients, individualizing treatment and reducing the types and quantities of medications as far as possible.

The common problem of frailty in the elderly population often leads to adverse effects, such as falls, disability and high hospitalization rates. Adverse outcomes in elderly patients with multimorbidity were found to occur at a mean rate of 13.9% with deep vein thrombosis being the most frequent at 42.4%. Regression analysis showed that frailty was a predictor of adverse health outcomes in elderly patients with multimorbidity (OR: 1.496; *p* < 0.01). A study showed that frailty was a significant predictor of future falls in hospitalized patients and due attention to frailty may lead to lowering the risk of falls [40]. Frailty has also been shown to be a high-risk predictive state for a series of adverse health outcomes [13]. For example, Cesari found that the 3-year cumulative mortality rate of frail elderly patients was 6 times that of non-frail patients [41] and a retrospective analysis by Ates Bulut et al*.* found a 33.6% fall rate in patients with comorbid frailty [42]. All the above studies suggest that frailty is directly related to the prognosis of patients with multimorbidity and appropriate interventions should be taken to prevent the occurrence of adverse events. There is great variation in current prevention and intervention measures for frailty with most targeted interventions being implemented for a specific ward. For example, 11 members of the Willson research team, including staff from nursing, medical, geriatrics, information technology, physical therapists and other related departments, developed customized care plans for patients who were debilitated after surgery. The completion rate for specific care instructions increased significantly, including preventive measures for aspiration (50.0% vs. 26.9%; *p* < 0.001) and for delirium (52.1% vs. 30.7%; *p* < 0.001). Multivariate analysis showed that postoperative complications were significantly reduced after 30 days of intervention (OR: 0.532; *p* < 0.001) [26]. However, there is a large and varied number of elderly patients with chronic multimorbidity, requiring multilevel interdisciplinary collaboration. The current study was limited to exploring large data samples to assess the current status of frailty, adverse outcome events and their influencing factors. The aim was to provide a reference for future intervention and prevention directions which should be explored in future studies.

## Conclusions

The current epidemiological study assessed the relationship between frailty and pre-frailty in elderly patients with chronic multimorbidity and the occurrence of adverse outcomes. Risk factors for frailty were identified. The prevalence of frailty among elderly hospitalized patients was 27.2% and that of pre-frailty was 58.9%. The rate of adverse outcomes among elderly frail patients was 13.9%. Increasing age, low BMI, low education level, lack of exercise, increased numbers of drug types and increased numbers of chronic diseases were all influencing factors for the occurrence of frailty. Frailty showed a higher morbidity in elderly patients with multimorbidity. Future research on the prediction, prevention and intervention of frailty to reduce the occurrence of adverse outcomes is warranted.

## Data Availability

The datasets used and/or analysed during the current study are available from the corresponding author on reasonable request.

## Abbreviations

BMI: Body Mass Index
FI: Frailty index
FRAIL: Fatigue, Resistance, Ambulation, Illnesses & Loss of Weight
CHD: Coronary Heart Disease
CHF: Chronic heart failure
COPD: Chronic obstructive pulmonary disease
CVD: Cerebral vascular diseases
CKD: Chronic kidney disease
CGD: Chronic gastrointestinal disease
CLD: Chronic liver disease
KOA: Knee osteoarthritis
DVT: Deep vein thrombosis
ICD-10: International Statistical Classification of Diseases and Related Health Problems, Tenth Revision
